# Sagittal spino-pelvic alignment in adults: The Wakayama Spine Study

**DOI:** 10.1371/journal.pone.0178697

**Published:** 2017-06-06

**Authors:** Yoshiki Asai, Shunji Tsutsui, Hiroyuki Oka, Noriko Yoshimura, Hiroshi Hashizume, Hiroshi Yamada, Toru Akune, Shigeyuki Muraki, Ko Matsudaira, Hiroshi Kawaguchi, Kozo Nakamura, Sakae Tanaka, Munehito Yoshida

**Affiliations:** 1 Department of Orthopedic Surgery, Wakayama Medical University, 811–1 Kimiidera, Wakayama City, Wakayama, Japan; 2 Department of Medical Research and Management for Musculoskeletal Pain, 22nd Century Medical and Research Center, Faculty of Medicine, The University of Tokyo, 7-3-1 Hongo, Bunkyo-ku, Tokyo, Japan; 3 Department of Joint Disease Research, 22nd Century Medical and Research Center, Faculty of Medicine, The University of Tokyo, 7-3-1 Hongo, Bunkyo-ku, Tokyo, Japan; 4 Rehabilitation Services Bureau, National Rehabilitation Center for Persons with Disabilities, 4–1 Namiki, Tokorozawa City, Saitama, Japan; 5 Japan Community Health Care Organization Tokyo Shinjuku Medical Center, 5–1 Tsukudocyo, Shinjuku-ku, Tokyo, Japan; 6 Department of Orthopedic Surgery, Faculty of Medicine, The University of Tokyo, 7-3-1 Hongo, Bunkyo-ku, Tokyo, Japan; University of Virginia, UNITED STATES

## Abstract

**Objectives:**

To establish the normal values of spino-pelvic alignment and to clarify the effect of age-related changes using large, community-based cohorts.

**Methods:**

In this study, data from 1461 participants (466 men, 995 women) were analyzed. On lateral standing radiographs, the following parameters were measured: thoracic kyphosis (TK), lumbar lordosis (LL), pelvic tilt (PT), pelvic incidence (PI), and C7 sagittal vertical axis (SVA). All values are expressed as the mean±standard deviation. The Spearman rank correlation coefficient was used to examine correlations between variables of spino-pelvic parameters. Finally, we analyzed the relationship between age and spino-pelvic parameters. Therefore, we entered values for the body mass index (BMI), SVA, TK, and PI-LL into a multiple regression model to adjust for potential confounding factors.

**Results:**

The SVA, TK, and PT increased with age, and LL decreased with age. Regarding sex differences, the TK was statistically significantly larger in men than in women, and LL, PT, and PI were statistically significantly smaller in men than in women. Correlation coefficients between the SVA and TK, between the SVA and PI-LL, and between TK and PI-LL were none, strong, and weak, respectively. Results of multiple regression analysis between age and spino-pelvic parameters showed that the standardized partial regression coefficients for the SVA, TK, and PI-LL were 0.17, 0.30, and 0.23, respectively, in men and 0.29, 0.32, and 0.23, respectively, in women.

**Conclusions:**

We found that all parameters were significantly associated with age in men and women. The SVA, TK, and PT increased with age, and LL decreased with age. Results of multiple regression analysis also demonstrated that the SVA, TK, and PI-LL are related to age. Indeed, the PI-LL value increased with age. In this study, a more excessive PI-LL mismatch was shown, indicating an increased risk of spinal malalignment. Differences in the absolute values of spino-pelvic parameters in each sex were small yet statistically significant. Thus, further study should be performed to corroborate this finding.

## Introduction

Sagittal spino-pelvic malalignment is one of the most prevalent disorders of the aging spine. Sagittal malalignment concerns are reflected in reports of flat back syndrome, which is an iatrogenic malalignment after spinal instrumentation that results in persistent lower back pain [1–4]. The sagittal curvature of the spine and pelvis balance each other to maintain a stable posture and horizontal gaze. Once the sagittal alignment is abnormal, more energy is required so that the body can remain balanced without external support [5]. Glassman et al. reported that positive sagittal balance was significantly related to clinical symptoms and health-related quality of life in patients with adult spinal deformity [6]. In addition, patients with kyphosis often complain of decreased walking ability and an increased propensity of falling, thereby resulting in weaker back extensor strength and poorer balance as well as heartburn due to gastroesophageal reflux disease, dysphasia, and respiratory symptoms [7–9]. Therefore, abnormal sagittal spinal alignment should be restored to normal. In previous studies, the C7 plumb line was used to measure sagittal global alignment [10–14]. The C7 sagittal vertical axis (SVA) is measured as the distance from the C7 plumb line to the posterosuperior endplate of the sacrum. The C7 plumb line has been used by previous authors to evaluate possible changes in sagittal spinal global alignment that occur with age. Increasing age was shown to correlate with increasingly anterior positions of the C7 plumb line [10, 11, 15]. Fon et al. [16] and Schwab et al. [15] proposed that the incidence of thoracic kyphosis (TK) increases with age. Youngbae et al. [17] hypothesized that the increase in TK is a fundamental change that occurs during aging. However, other studies [10, 11] did not support this hypothesis. Gelb et al. [11] reported that TK did not correlate with age in healthy older individuals, despite significant losses in lumbar lordosis (LL) and SVA. The pelvic incidence (PI) is unique to each individual and independent of the spatial alignment of the pelvis. The PI reflects the anatomy of the pelvis and does not change with pelvic or spine positioning [10, 18–21]. PI is an important anatomic parameter that reflects the anatomic configuration of the pelvis and greatly affects sagittal spino-pelvic alignment (SSPA). PI-LL has been considered to be a useful indicator in intraoperative planning of lumbar deformity operation [19, 22, 23]. PI-LL is significantly correlated with clinical parameters. Schwab et al. recommend that PI-LL should be corrected to less than 10° to achieve successful, harmonious spino-pelvic realignment in corrective operation of spinal deformity [19].

Recently, it has become possible to achieve optimal spinal alignment with the development of spinal operation techniques. There have been some reports regarding the normal values of SSPA [11, 24–27]. In addition, the optimal postoperative SSPA can be evaluated during preoperative surgical planning of spinal realignment based on these reported parameters [19, 28]. However, most of our previous studies were performed using Caucasian populations in the United States and European countries. The effect of ethnicity on skeletal growth has been demonstrated by previous studies [29, 30]. Age and sex are also reported to be associated with spino-pelvic alignment [31, 32]. Recently, some studies were conducted to evaluate the normal SSPA in Asian populations [33–35]. However, the number of participants was small, and only young adults were evaluated in these studies. The present study sought to establish the normal values of spino-pelvic alignment and to clarify the impact of age-related changes using large, community-based cohorts.

## Materials and methods

### Participants

Under the approval of our institutional review board, the present study, titled the Wakayama Spine Study, was performed with a sub-cohort of the third visit of the Research on Osteoarthritis/Osteoporosis Against Disability (ROAD) study, which was initiated as a nationwide, prospective study of bone and joint diseases in population-based cohorts. A detailed profile of the ROAD cohort has been previously reported [36, 37]. In brief, subjects included participants of the third visit of the ROAD study, which began in 2012 and completed in 2013. In addition to the former participants, inhabitants of the mountainous and coastal areas in the Wakayama prefecture who were willing to participate in the ROAD survey were also included in the third visit. Overall, 1575 individuals (513 men, 1062 women) participated in the third visit of the ROAD study. Among 1575 participants, 114 individuals who could not maintain a standing position while undergoing total lateral whole-spine radiography or had other disqualifiers were excluded. Finally, lateral whole-spine radiographs were available for 1461 participants (466 men, 995 women).

Participants were divided into five groups based on birth-year decade: (1) less than 50 years, (2) 50–59 years, (3) 60–69 years, (4) 70–79 years, and (5) 80 years and older. All individuals provided written informed consent.

### Radiographic evaluation

All participants underwent radiography. For each subject, standing lateral radiography of the whole spine and pelvis was taken using 40-inch film. Each radiograph was aligned such that the edge of the film was the reference for vertical alignment. As described previously [34], participants were instructed to stand in a comfortable position, with their hips and knees fully extended. The arms were flexed with the hands resting on supports at the level of their shoulders.

On the radiographs, the following parameters were measured: TK (the Cobb angle from the upper endplate of T2 to the lower endplate of T12) [16], LL (the Cobb angle from the upper endplate of L1 to the lower endplate of S1) [23], pelvic tilt (PT) (the angle between the line connecting the midpoint of the sacral plate to the axis of the femoral heads and the vertical axis) [19], PI (the angle between the line perpendicular to the sacral plate at its midpoint and the line connecting this point to the axis of the femoral heads) [24], and SVA (the horizontal distance from the C7 plumb line originating at the middle of the C7 vertebral body to the posterior superior endplate of S1) [19].

### Statistical analysis

Statistical analyses were performed using JMP (version 8; SAS Institute Inc., Cary, NC). All values are expressed as the mean ± standard deviation (SD). The Wilcoxon signed-rank test was used to analyze the differences in spinal and pelvic parameters between men and women. The Spearman rank correlation coefficient (*r*) was used to examine correlations between variables of spino-pelvic parameters. The Spearman correlation coefficient was interpreted as follows: <0.3: none; 0.31–0.5: weak; 0.51–0.7: strong; 0.71–0.9: very strong; and >0.9: excellent. Finally, we analyzed the relationship between age and spino-pelvic parameters. Therefore, we entered values for the body mass index (BMI), SVA, TK, and PI-LL into a multiple regression model to adjust for potential confounding factors. The variance inflation factor (VIF) was used to check for multicollinearity in the model. The level of statistical significance was set at 0.05.

## Results

Radiographic studies were completed for 1461 participants (466 men, 995 women) whose age range was 19–94 years (mean age: men, 66.3 ± 13.8 years; women, 65.2 ± 12.5 years). The average BMI was 23.0 ± 3.5 kg/m^2^ (Table 1).

**Table 1:** Participants’ demographic data.

	Total	Men	Women
**Number of participants**	**1461**	**466**	**995**
**Age strata (years)**			
≤49	170	56	114
50–59	256	75	181
60–69	418	124	294
70–79	407	123	284
≤80	210	88	122
**Demographic characteristics**			
Age (years)	65.6 ± 13.0	66.3 ± 13.8	65.2 ± 12.5
Height (cm)	156.0 ± 9.1	164.7 ± 7.3	151.8 ± 6.7
Weight (kg)	56.2 ± 11.1	64.2 ± 11.4	52.4 ± 8.7
BMI (kg/m^2^)	23.0 ± 3.5	23.6 ± 3.4	22.8 ± 3.5

Values are presented as the mean ± standard deviation.

BMI, body mass index.

The mean value and SD of spino-pelvic parameters are listed in Tables 2 and 3. The SVA, TK, and PT increased with age, and LL decreased with age. Regarding sex differences, TK was significantly larger in men than in women, and LL, PT, and PI were significantly smaller in men than in women.

**Table 2:** Spinal parameters.

	Sagittal vertical axis (mm)					Thoracic kyphosis (°)					Lumbar lordosis (°)				
Age	Men		Women			Men		Women			Men		Women		
(years)	Mean	SD	Mean	SD	p value	Mean	SD	Mean	SD	p value	Mean	SD	Mean	SD	p value
≤**49**	**-11.0**	**22.9**	**-18.8**	**24.6**	0.025^*^	**33.5**	**7.2**	**33.1**	**9.3**	**0.757**	**47.9**	**9.1**	**52.0**	**10.3**	0.013^*^
**50–59**	**4.7**	**30.7**	**-8.2**	**25.6**	<0.001^*^	**36.0**	**9.2**	**34.2**	**10.6**	**0.192**	**45.2**	**9.5**	**48.9**	**10.4**	0.007^*^
**60–69**	**8.2**	**35.0**	**1.8**	**32.7**	**0.073**	**37.8**	**9.1**	**36.2**	**11.4**	**0.193**	**45.2**	**11.8**	**46.9**	**12.8**	**0.057**
**70–79**	**13.9**	**39.2**	**24.2**	**43.1**	0.011^*^	**41.6**	**11.8**	**38.9**	**12.7**	0.038^*^	**45.7**	**13.4**	**43.6**	**14.2**	**0.110**
≤**80**	**39.1**	**54.3**	**51.6**	**57.9**	**0.101**	**40.3**	**12.3**	**44.2**	**17.8**	**0.122**	**39.3**	**15.9**	**38.6**	**19.1**	**0.922**
**Total**	**12.7**	**41.3**	**10.1**	**43.4**	**0.062**	**38.5**	**10.7**	**37.2**	**12.8**	0.022^*^	**44.5**	**12.7**	**45.9**	**14.0**	0.019^*^

SD, standard deviation

^*^Significant difference between men and women (p<0.05, Wilcoxon signed-rank test)

**Table 3:** Pelvic parameters.

	Pelvic tilt (°)					Pelvic incidence (°)				
Age	Men		Women			Men		Women		
(years)	Mean	SD	Mean	SD	p value	Mean	SD	Mean	SD	p value
≤**49**	**11.5**	**6.9**	**14.5**	**6.7**	0.006^*^	**46.0**	**9.9**	**51.0**	**10.8**	0.002^*^
**50–59**	**14.4**	**6.6**	**15.8**	**8.0**	**0.161**	**47.7**	**9.6**	**49.8**	**10.5**	**0.174**
**60–69**	**15.5**	**6.8**	**18.1**	**8.5**	0.001^*^	**48.3**	**9.9**	**50.6**	**10.4**	0.028^*^
**70–79**	**16.0**	**7.5**	**23.0**	**10.3**	<0.001^*^	**47.5**	**9.3**	**52.7**	**10.7**	<0.001^*^
≤**80**	**19.7**	**8.4**	**25.2**	**10.4**	<0.001^*^	**48.1**	**10.9**	**51.6**	**11.4**	0.021^*^
**Total**	**15.8**	**7.6**	**19.5**	**9.7**	<0.001^*^	**47.7**	**9.9**	**51.2**	**10.8**	<0.001^*^

SD, standard deviation

^*^ p<0.05

The correlation coefficients (*r*) between the SVA and TK, between SVA and PI-LL, and between TK and PI-LL were none (0.12), strong (0.54), and weak (-0.33), respectively (Table 4).

**Table 4:** Correlation matrix among the spino-pelvic parameters.

Parameter	SVA	TK	PI-LL
SVA	1		
			
TK	0.12	1	
	<0.001^*^		
PI-LL	0.54	-0.33	1
	<0.001^*^	<0.001^*^	

Upper line, correlation coefficient; lower line, p-value. SVA, sagittal vertical axis; TK, thoracic kyphosis; PI, pelvic incidence; LL, lumbar lordosis

^*^Significant correlation between the parameters (p<0.05)

Table 5 shows the results from multiple regression analysis, after adjusting for various confounding factors. The VIF values in men for BMI, SVA, TK, and PI-LL were 1.04, 1.76, 1.45, and 2.13, respectively; those in women were 1.02, 2.27, 1.42, and 2.52, respectively. However, none of the VIF values exceeded 10, which indicates that there was no collinearity in the model [38]. On the basis of the results of this model, we found that all parameters were significantly associated with age in men and women. The standardized partial regression coefficients of SVA, TK, and PI-LL were 0.17, 0.30, and 0.23, respectively, in men and 0.29, 0.32, and 0.23, respectively, in women. PT had a high collinearity with other parameters, and it was excluded from Tables 4 and 5.

**Table 5:** Results of multiple regression analysis between age and spino-pelvic parameters.

	Men		Women	
	Standardized partial regression coefficient	p value	Standardized partial regression coefficient	p value
**SVA**	**0.17**	0.0015*	**0.29**	<0.001*
**TK**	**0.30**	<0.001*	**0.32**	<0.001*
**PI-LL**	**0.23**	0.0001*	**0.23**	<0.001*

SVA, sagittal vertical axis; TK, thoracic kyphosis; PI, pelvic incidence; LL, lumbar lordosis

^*^Significant correlation between age and parameters (p<0.05)

## Discussion

In this study, the SVA, TK, and PT increased with age, and LL decreased with age. In addition, the rate of increase in TK and decrease in LL was larger in women than in men, although the mean values of these parameters were within the generally accepted normal ranges [39–41].

Fon et al. reported that the degree of kyphosis increased with age, and the rate of increase was higher in women than in men [16]. This observation has been widely observed since increased TK is often related to osteoporotic compression wedging of the vertebrae, as well as to the degenerative change of intervertebral discs and decreased strength of back extensor muscles in the aged spine [42–51]. This degenerative change can also contribute to decreased LL [52]. Gelb et al. investigated 100 asymptomatic middle and older aged volunteers, and they found a correlation among the SVA, LL, and age [11]. These findings were supported by Hammerberg and Wood, whose study surveyed 50 asymptomatic volunteers aged 70–85 years [10]. The aforementioned correlation between spino-pelvic parameters and age may explain physiological aging of the spine. The center of gravity line moves forward in relation to increasing age [15], which may result in pain, functional disability, and loss of horizontal gaze due to the stooped posture. In an attempt to correct this position that interferes with the social standard of maintaining a horizontal gaze, the pelvis should be tilted backward [53].

The impact of sex on spino-pelvic parameters remains controversial. Vialle et al. reported significant differences in LL and PI between male and female subjects [27]. In addition, Zhu et al. found a significant sex difference in LL [34]. Conversely, other researchers did not demonstrate significant sex differences in any spino-pelvic parameter [32, 33, 54]. Although there were statistically significant differences in TK, LL, PT, and PI between men and women in the current study, the difference in the mean value of each parameter was quite small. Additionally, the individual variations were much larger than were the sex differences. When considering clinically important differences, further study should be performed to corroborate this finding. Recently, there have been some reports to support racial differences in sagittal spino-pelvic parameters [30], and most of them have exaggerated the smaller PI and LL in Asian populations than in Caucasian populations [33–35]. However, our cohort did not have a significantly smaller PI than did the Caucasian population, which is consistent with the Japanese epidemiological study by Takeda et al.[55], which reported a PI of 55.8 ± 10.6. There may be regional differences in sagittal spino-pelvic parameters as well.

Therefore, there must be strong correlations among spino-pelvic parameters. Legaya et al. reported that PI is a fundamental pelvic parameter for three-dimensional regulation of spinal sagittal curves, and it correlates with LL [24]. In addition, Mac-Thiong et al. demonstrated a moderate correlation (0.3 ≤ *r* < 0.5) between TK and LL [56]. Our results also suggested strong correlations between the SVA and PI-LL, as well as weak correlations between TK and PI-LL. To achieve harmonized, spino-pelvic alignment in surgical planning for spinal deformity, the PI-LL value was used to determine the amount of correction needed. In a recent study, a more excessive PI-LL mismatch was shown to indicate an increased risk of spinal imbalance [23]. Results of multiple regression analysis also demonstrated that the SVA, TK, and PI-LL are related to age. Indeed, the PI-LL value increased with age.

A longitudinal study would be required to assess the age-related changes of the sagittal spino-pelvic parameters accurately. Moreover, evaluation of the alignment of the cervical spine and/or lower extremities should be included since they also definitively show age-related changes and affect spino-pelvic alignment.

Recently, more researchers have focused on spino-pelvic alignment because of the increasing number of adult patients in the aging society with back pain related to spinal malalignment. However, to the best of our knowledge, the current study, compared to previous studies, makes use of the largest cohort (more than 1,500 volunteers) from general populations with a wide range of ages. In doing so, we were able to better understand age-related and sex-related normal values of spino-pelvic sagittal alignment, although the study was performed in limited districts. Thus, we believe that this study’s findings may help improve the treatment of patients with adult spinal deformity.

## Conclusions

We found that all parameters were significantly associated with age in men and women. The SVA, TK, and PT increased with age, and LL decreased with age. Additionally, a more excessive PI-LL mismatch was shown to indicate an increased risk of spinal malalignment. Results of multiple regression analysis also demonstrated that the SVA, TK, and PI-LL are related to age. Indeed, the PI-LL value increased with age.
